# rt269I Type of Hepatitis B Virus (HBV) Polymerase versus rt269L Is More Prone to Mutations within HBV Genome in Chronic Patients Infected with Genotype C2: Evidence from Analysis of Full HBV Genotype C2 Genome

**DOI:** 10.3390/microorganisms9030601

**Published:** 2021-03-15

**Authors:** Hyein Jeong, Dong Hyun Kim, Yu-Min Choi, HyeLim Choi, Donghyun Kim, Bum-Joon Kim

**Affiliations:** 1 Department of Biomedical Sciences, Microbiology and Immunology, and Liver Institute, College of Medicine, Seoul National University, Seoul 03080, Korea; zzzzihye@snu.ac.kr (H.J.); shady6233@snu.ac.kr (D.H.K.); cym486486@snu.ac.kr (Y.-M.C.); 2 Department of Biomedical Sciences, and Microbiology and Immunology, College of Medicine, Seoul National University, Seoul 03080, Korea; helenchoi501@gmail.com (H.C.); biologokim@snu.ac.kr (D.K.)

**Keywords:** rt269L-type hepatitis B virus (HBV), rt269I, nonsynonymous (NS) mutation, genotype C2

## Abstract

Recently, it has been reported that the rt269I type of hepatitis B virus (HBV) polymerase (Pol) versus the rt269L type is more significantly related to lower viral replication and HBeAg negative infections in chronic hepatitis B (CHB) patients of genotype C2. In this study, we compared mutation rates within HBV genomes between rt269L and rt269I using a total of 234 HBV genotype C2 full genome sequences randomly selected from the HBV database (115 of rt269L and 119 of rt269I type). When we applied the Benjamini and Hochberg procedure for multiple comparisons, two parameters, dN and d, at the amino acids level in the Pol region were significantly higher in the rt269I type than in the rt269L type. Although it could not reach statistical significance from the Benjamini and Hochberg procedure, nonsynonymous (NS) mutations in the major hydrophilic region (MHR) or “a” determinant in the surface antigens (HBsAg ORF) related to host immune escape or vaccine escape are more frequently generated in rt269I strains than in rt269L. We also found that there are a total of 19 signature single nucleotide polymorphisms (SNPs), of which 2 and 17 nonsynonymous mutation types were specific to rt269L and rt269I, respectively: Of these, most are HBeAg negative infections (preC-W28*, X-V5M and V131I), lowered HBV DNA or virion production (C-I97F/L, rtM204I/V) or preexisting nucleot(s)ide analog resistance (NAr) (rtN139K/H, rtM204I/V and rtI224V) or disease severity (preC-W28*, C-I97F/L, C-Q182K/*, preS2-F141L, S-L213I/S, V/L5M, T36P/S/A, V131I, rtN139K/H, rtM204I/V and rtI224V). In conclusion, our data showed that rt269I types versus rt269L types are more prone to overall genome mutations, particularly in the Pol region and in the MHR or “a” determinant in genotype C2 infections and are more prevalent in signature NS mutations related to lowered HBV DNA replication, HBsAg and HBeAg secretion and potential NAr variants and hepatocellular carcinoma (HCC), possibly via type I interferon (IFN-I)-mediated enhanced inflammation. Our data suggest that rt269L types could contribute to liver disease progression via the generation of immune escape or enhanced persistent infection in chronic patients of genotype C2.

## 1. Introduction

Despite the availability of an effective vaccine, hepatitis B virus (HBV) infection is still a high-risk global health issue; with more than 240 million people being chronic carriers of the virus, approximately 786,000 patients annually die worldwide due to hepatitis B virus (HBV)-related diseases, including cirrhosis and hepatocellular carcinoma (HCC) [1]. HBV infection is endemic in South Korea, with the prevalence of hepatitis B virus surface antigen (HBsAg) positivity being 3.4% among men and 2.6% among women on the basis of the Korean National Health and Nutrition Survey of 2011 [2].

HBV has an enveloped and partially double-stranded DNA virus and preferentially replicates in hepatocytes with an incomplete double-stranded DNA genome of approximately 3.2 kb in length; it contains four overlapping open reading frames (ORFs): surface antigens (S), core proteins (C), polymerase (Pol), and X proteins (X) [3]. HBV reverse transcriptase (RT) lacks proofreading ability, leading to HBV mutations that occur at a 10-fold higher frequency than other DNA viruses [4]. This compromises antiviral therapy with nucleos(t)ide analogs (NAs) and affects disease progression via persistent infections.

On the basis of an 8% divergence in HBV genome sequences, HBV has been characterized into 10 genotypes as A–J [5]. There is increasing evidence that specific HBV genotypes may play significant roles in the development of different disease profiles during chronic hepatitis B (CH) infection [6,7]. Among the 10 genotypes, genotypes B and C are widespread in Asia, but two genotypes lead to distinctly different clinical outcomes [8]. Compared to genotype B, genotype C, particularly C2, showed higher HBV replication capacity, a higher tendency of chronicity and more frequently developed into liver cirrhosis (LC) and hepatocellular carcinoma (HCC) [9]. In addition, incomplete response to interferon (IFN) therapy and higher levels of mutations were also reported in genotype C infection. Notably, an extraordinary prevalence of virulent genotype C2 has been reported in South Korea [10,11,12]. Furthermore, the presence of a distinct immune response against HBV proteins in the Korean population can lead to the generation of unique HBV variants that are rarely encountered in other areas, resulting in distinct clinical manifestations in Korean chronic patients [13]. Indeed, several unique types of HBV mutations related to the progression of liver disease encountered in other areas have been found in South Korea [14,15,16,17,18,19,20,21,22,23,24,25,26,27,28,29]. To date, higher mutation rates and the presence of distinct mutations found in genotype C2 infections remain largely unknown. However, recently, we have reported that the presence of two HBV Pol RT polymorphisms, rt269L and rt269I, distinct only in HBV genotype C, could play a very pivotal role in viral phenotypes, clinical outcomes, and worse responses to IFN therapy distinct in genotype C2 infections. In particular, we showed that rt269I-type infection versus rt269L-type infection leads to enhanced mitochondrial stress-mediated type I interferon (IFN-I) production, resulting in HBV e antigen (HBeAg)-negative infections by generating preC mutations at 1896 (G to A). Therefore, in this study, we hypothesized that HBeAg seroconversion, frequently found in patients infected with the rt269I type of genotype C2, could lead to increased genome mutations in the rt269I type versus the rt269L type by inducing immune responses against HBV. To address this issue, we compared overall mutation rates and signature mutation SNPs specific to the respective RT polymorphisms rt269L and rt269I using a total of 234 HBV genotype C2 full genome sequences retrieved from a public database (Hepatitis B Virus Database: https://hbvdb.ibcp.fr/HBVdb/ (accessed on 14 March 2021)), of which 115 and 119 genome sequences were from rt269L and rt269I types, respectively.

## 2. Materials and Methods

### 2.1. HBV Sequence Acquisition and Processing

First, a total of 252 HBV genotype C2 sequence data were downloaded from the Hepatitis B Virus Database (https://hbvdb.ibcp.fr/HBVdb/ (accessed on 14 March 2021)) [30]. The accession numbers of downloaded sequences are shown in Supplementary Table S1. Of the 252 HBV sequences, 234 except for 18 strains identified as subgenotypes, C3 and C4, by our full genome-based phylogenetic analysis were finally determined as genuine genotype C2 strains. For the analysis of mutation rates, each of the five genes (preS, S, C, P, and X) was extracted and aligned separately using the MEGA X program (USA). Every gap and insert were deleted and aligned, so the whole length of the sequences was fit to 3215 bp. All the sequence sites were numbered for the *EcoR*1 restriction site to be located as a first nucleotide (nt). For the purpose of the study, every sequence was checked at the 269th codon on reverse transcriptase to classify the L type (rt269L) and I type (rt269I).

### 2.2. HBV Phylogenetic Analysis and Intergenotypic Recombination Analysis

A phylogenetic analysis based on entire sequences of HBV full genome sequences (3215 bp) was performed for further subgenotype separation of 252 HBV designated genotype C2 from HBVdb [30]. The analysis was conducted with five genotype reference strains (M57663 (A), AB100695 (B), X02496 (D), AB106564 (E), and X75663 (F)) and thirteen recently described subgenotype reference strains [31] (including C1 (AB074047, EU306686, GQ924619), C2 (KM99991), C3 (AB115417, AB115418, AY167091), C4 (EU939624, AY206374, AB675675), and C5 (GQ924620, GQ924657, AP011100) obtained from GenBank. A phylogenetic tree was inferred by the neighbor-joining method using Tamura–Nei genetic distance model and 100 replicates of bootstrap tests were conducted [32,33,34]. HBV genotype B sequences were used as the outgroup (accession No. D00329). The intergenotypic recombination of sequences of the 234 strains finally identified as genuine subgenotype C2 were analyzed with five genotype reference strains (M57663 (A), AB100695 (B), X02496 (D), AB106564 (E), and X75663 (F)) using DualBrothers recombination detection software [35].

### 2.3. Analysis of Mutation Rates Based on the HBV Full Genome and 5 HBV Regions (preS1, S, preC/C, X and Pol)

Genetic diversity, including the mean genetic distance (d), the number of synonymous substitutions per synonymous site (dS), and the number of nonsynonymous substitutions per nonsynonymous site (dN), was calculated for each reference strain of 234 HBV sequences finally determined as genotype C2 infections. The mean genetic distance was a measure of the accumulated number of nucleotide or codon differences per locus from the consensus sequence. The consensus sequence was defined as the calculated order of most frequent residues, either nucleotide or amino acid, found at each position in a sequence alignment. The consensus sequence was differently calculated in 115 rt269L and 119 rt269I strains, but if the consensus sites obtained in rt269L and rt269I were different at a nucleotide or amino acid position, the consensus at the position included both of them. Exceptionally, adenine substitution at 1762 nt and guanine substitution at 1764 nt, which have been widely accepted as wild-type sequences in the basal core promoter (BCP) [36], were defined as wild type. The consensus sequences were calculated in each open reading frame (PreS, S, preC/C, X, and Pol). The number of amino acid variants was calculated as the sum of those in each ORF, taking into account the partially overlapping ORFs of HBV.

The genetic distances at the nucleotide level were calculated under the Tamura three-parameter model, taking into account the differences in transitional and transversional rates and G+C-content bias. Meanwhile, the genetic distances at the amino acid level were calculated under the Jones–Taylor–Thorton (JTT) model. The number of both nucleotide and amino acid variants was calculated. The dS and dN were calculated under the modified Nei–Gojobori model with Jukes–Cantor correction. All genetic diversity factors were tested using MEGA X software (USA) [37].

### 2.4. Statistical Analysis

The results of continuous variables are expressed as medians and standard deviations. The genetic diversity between rt269L and rt269I was compared using independent sample *t*-tests. Mutation rate of signature nonsynonymous mutation is defined as the proportion of the number of strains among each group. The signature of nonsynonymous mutation between rt269L and rt269I was compared using chi-squared test. The statistical analysis was performed using GraphPad Prism 8.0 software (GraphPad, La Jolla, CA, USA). For the analysis of signature nonsynonymous mutations, continuous variables were tested using the chi-squared test using SPSS 25.0.0.0 software (SPSS Inc., Chicago, IL, USA). Significant differences (* *p <* 0.05, ** *p* < 0.01, *** *p* < 0.001) among the different groups are shown in the related figures, and the bar plot data are presented as the means ± s.e.m. of each group. Additionally, multiple comparison using Benjamini–Hochberg correction (false discovery rate (FDR)) was also performed by R 3.5.2 software and the significant values (^a^ *q* < 0.05, ^b^ *q* < 0.01, ^c^ *q* < 0.001) are shown in the related tables [38,39].

## 3. Results

### 3.1. Genotype Determination of HBV Full Genome Sequences via Phylogenetic Analysis

**Figure 1:**
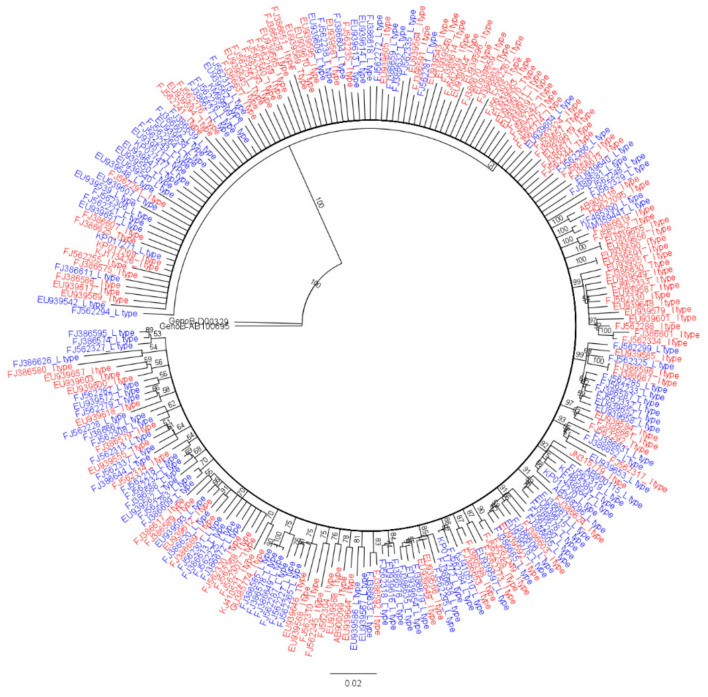
Genotype determination of selected HBV genomes via phylogenetic analysis. Phylogenetic tree of a total of 234 HBV sequences belonging to genotype C2 was conducted by the neighbor-joining method using Tamura–Nei genetic distance model. A total of 100 replicate bootstrap tests were conducted using a genotype B sequence as an outgroup. rt269L and rt269I sequences labeled “L” and “I” at the end of accession number.

To analyze overall genome mutation rates and signature SNPs between HBV genotype C2 strains of two RT polymorphisms, rt269L and rt269I, 252 HBV strains belonging to genotype C2 were randomly selected from the HBV database HBVdb (http://hbvdb.ibcp.fr (accessed on 14 March 2021)) [30]. Since 18 (2 strains of rt269L and 16 strains of rt269I) of 252 strains designated genotype C2 in HBVdb were identified into subgenotypes, C3 and C4, by our phylogenetic analysis based on a recent study newly defining HBV genotypes and subgenotypes [31,40,41] (data not shown), we finally determined 234 HBV strains (92.9%) as genotype C2, which were used for our genome study. They were further separated into 115 rt269L types (49.1%) encoding leucine and 119 rt269I types (50.9%) encoding isoleucine at the 269th codon of RT via sequence comparison (Figure 1 and Supplementary Table S1).

### 3.2. Comparison of Genetic Diversity of the HBV Full Genome between rt269L and rt269I

**Figure 2:**
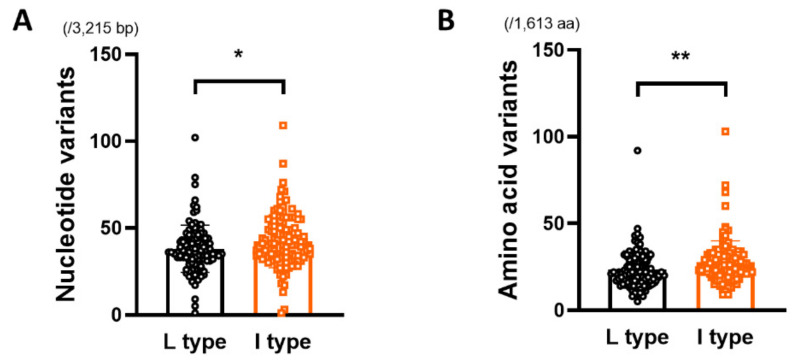
Comparison of genetic diversity of the HBV full genome between rt269L and rt269I. Comparison of the number of (**A**) nucleotide and (**B**) amino acid variants within the HBV full genome between rt269L and rt269I. Significant differences (* *p* < 0.05, ** *p* < 0.01) analyzed by independent samples *t*-test between the groups are shown in the related figures. The bar plot data are presented as the mean ± s.e.m. of each group.

The genome diversity of the HBV genome between rt269L and rt269I, including the mean genetic distance (d), the number of synonymous substitutions per synonymous site (dS), and the number of nonsynonymous substitutions per nonsynonymous site (dN), was calculated for each HBV strain using independent sample *t*-test. As HBV partially overlaps four open reading frames encoding the surface antigen, nucleocapsid, X protein (X) and polymerase (Pol), we separately estimated the total number (1613 aa) of amino acid mutations in the presurface (PreS) domain, S protein, X, precore/core (PreC/C), and Pol coding regions of the HBV genome. Our statistical analysis using *t*-test showed that the numbers of nucleotide and amino acid variants in the HBV full genome of the rt269L type (38.03 ± 13.65/3215 bp and 23.43 ± 10.75 variants/1613 aa, respectively) were significantly lower than those of the rt269I type (42.34 ± 15.17/3215 bp and 27.68 ± 12.39/1613 aa variants, respectively) (* *p*= 0.0234 and ** *p* = 0.0056, respectively) (Table 1 and Figure 2).

### 3.3. Comparison of Genetic Diversity in the PreS, S, X, PreC/C and Pol Regions between rt269L and rt269I

**Figure 3:**
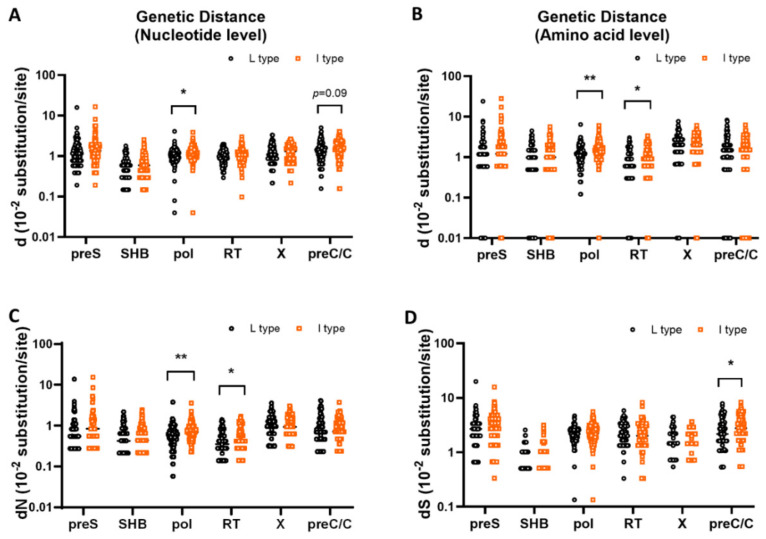
Comparison of genetic diversity in the PreS, S, X, PreC/C and Pol regions between rt269L and rt269I. Comparison of genetic distance at the nucleotide and amino acid levels (**A**,**B**), dN (the number of nonsynonymous substitutions per nonsynonymous site) (**C**), and dS (the number of synonymous substitutions per synonymous site) (**D**) within the PreS, S, X, PreC/C and Pol regions between rt269L and rt269I. Significant differences (* *p* < 0.05, ** *p* < 0.01) analyzed by independent samples *t*-test between the groups are shown in the related figures.

The genetic diversity within the PreS, S, X, PreC/C, and Pol regions, including the nucleotide and amino acid levels, was calculated for each strain in both rt269L and rt269I using the independent sample *t*-test. Our statistical analysis using the *t*-test showed that in three regions—the PreS, S, and X regions—there were no significant differences between rt269L and rt269I types in d at either the nucleotide or amino acid level, dN, and dS (*p* > 0.05). However, in the Pol region, dNS and d at both the nucleotide and amino acid levels were significantly lower in the rt269L type (0.61 ± 0.42, 1.09 ± 0.46, and 1.26 ± 0.79 ×10^−2^ substitutions/site, respectively) than in the rt269I type (0.78 ± 0.45, 1.24 ± 0.50, and 1.60 ± 0.86 ×10^−2^ substitutions/site, respectively) (** *p* = 0.0044, * *p* = 0.0154, and ** *p* = 0.0019 from *t*-test, respectively). In particular, in the RT region of HBV polymerase, dN and d at the amino acid level were significantly lower in the rt269L type (0.42 ± 0.32 and 0.88 ± 0.67 ×10^−2^ substitutions/site, respectively) than in the rt269I type (0.51 ± 0.35 and 1.06 ± 0.72 ×10^−2^ substitutions/site, respectively) (* *p* = 0.0327 and * *p* = 0.0465 from *t*-test, respectively). In the PreC/C region, dS and d at the nucleotide level were significantly or tended to be lower in the rt269L type (2.45 ± 1.62 and 1.38 ± 0.86 ×10^−2^ substitutions/site, respectively) than in the rt269I type (2.94 ± 1.62 and 1.56 ± 0.80 ×10^−2^ substitutions/site, respectively) (* *p* = 0.0212 and *p* = 0.0989 from *t*-test, respectively) but not different in dNS and d at the amino acid level (Table 1 and Figure 3). Moreover, when we applied the Benjamini and Hochberg procedure to adjust the raw *p*-values for multiple comparisons, notably, two parameters, dN and d, at the amino acids level in the Pol region still had significant difference, although most corrected *p*-values did not reach the statistical significance (Table 1). Together, our data indicated that rt269I types versus rt269L are more prone to overall genome mutations, particularly in the Pol region.

### 3.4. rt269I versus rt269L Is More Prone to NS Mutations in the MHR or “a” Determinant Region

**Figure 4:**
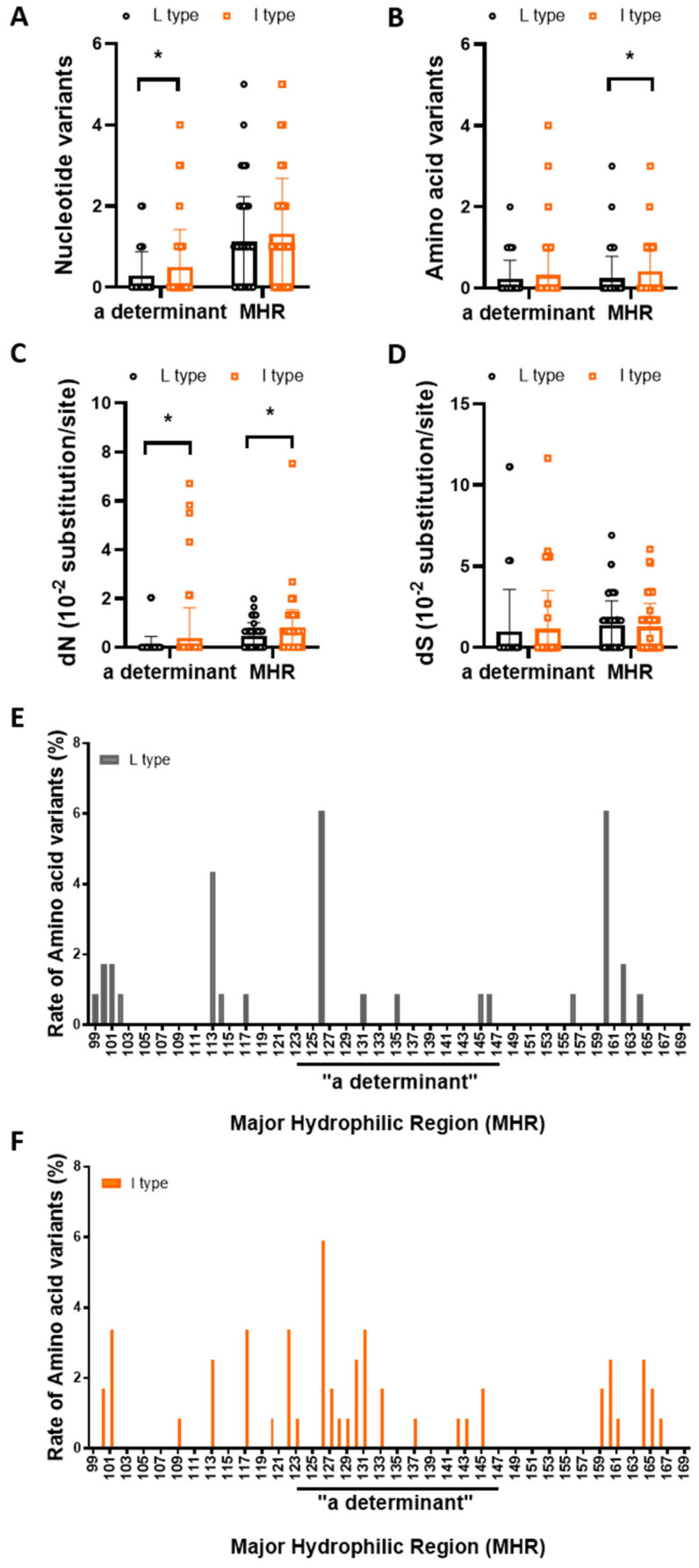
rt269I versus rt269L is more prone to NS mutations in the MHR or “a” determinant region. Comparison of the number of nucleotide and amino acid variants (**A**,**B**), dN (**C**), and dS (**D**) within the major hydrophilic region and the “a” determinant. Significant differences (* *p* < 0.05) analyzed by independent samples *t*-test between the groups are shown in the related figures. Prevalence of residue substitutions within MHR from rt269L (black bars) and rt269I (orange bars) (**E**,**F**). Each bar represents the rate of amino acid variants located on a given amino acid position.

The major hydrophilic region (MHR) ranges from residues 99 to 169 of HBsAg, and because it is probably composed of several B cell epitopes, mutations within the MHR, especially in the ‘‘a’’ determinant region (residues 124–147), can affect the antigenicity of HBsAg, resulting in the escape of the virus from neutralizing antibody responses and persistent viral infections [42]. The prevalence and pattern of MHR mutations between rt269L and rt269I strains were investigated (Table 2 and Figure 4). Our statistical analysis using *t*-test showed that the number of nucleotides in the “a” determinant and the amino acid variants in the MHR between rt269L and rt269I were significantly higher in rt269I (0.29 ± 0.59 and 0.50 ±0.92 ×10^−2^ variants in the “a” determinant, 0.24 ± 0.54 and 0.41 ± 0.69 ×10^−2^ variants in the MHR, respectively) than in rt269L (* *p* = 0.0311 and * *p* = 0.0399 from *t*-test, respectively). Further analysis showed that dS was not significantly different. dN in both MHR and “a” determinant were significantly lower in rt269L type (0.07 ± 0.38 and 0.38 ± 1.24 ×10^−2^ substitutions/site in “a” determinant, 0.48 ± 0.53 and 0.67 ± 0.87 ×10^−2^ substitutions/site in MHR, respectively) than in rt269I type (* *p* = 0.0109 and *p* = 0.0497 from *t*-test, respectively). However, all these differences between rt269L and rt269I shown by *t*-test did not reach statistical significance in multiple comparisons using Benjamini–Hochberg correction. Together, while it may not be so obvious, NS mutations in the MHR or “a” determinant in the S ORF related to host immune escape or vaccine escape are more frequently generated in rt269I strains than in rt269L, suggesting that the former is more likely to lead to persistent infection in chronic patients with genotype C2 than the latter.

### 3.5. Identification of Signature Nonsynonymous Mutations Specific to rt269L or rt269I

Next, we identified signature nonsynonymous mutations specific to rt269L or rt269I in the PreS, S, X, PreC/C and Pol regions, of which frequencies are significant or tend to be higher in rt269L or rt269I. The mutation rate of each signature nonsynonymous mutation was defined as the proportion of the number of strains among each group, and mutation rate between rt269L and rt269I was compared using chi-squared test. Thereafter, the resulting *p*-values were calculated by multiple comparison using Benjamini–Hochberg correction (FDR). A total of 19 types of signature nonsynonymous mutations were found in five HBV regions, PreS (three types), S (two types), X (four types), PreC/C (three types) and Pol regions (seven types). Of these, 2 and 17 nonsynonymous mutation types were specific to rt269L and rt269I, respectively (Table 3).

#### 3.5.1. PreS and S Region

HBV S ORF is composed of preS1, preS2 and S regions. NS mutations in these regions could lead to liver disease progression by escaping host immune responses and eliciting endoplasmic reticulum stress [43]. We found a total of five types of signature NS mutations in the S ORF (I84T/M/L/V and A90T/V in PreS1, F141L in PreS2, and I68T and L213I/S in S). Of these, the frequency in all four types but one rt269L signature type (I68T in the S region) was significantly or tended to be higher in rt269I HBV strains than in rt269L. Of the four rt269I signature types, I84T/M/L/V in PreS1 has been reported to be frequently found in HBV occult cases [15]. F141L in PreS2 has been reported to be significantly related to HCC progression in Korean chronic patients by inducing cell cycle progression by downregulating the p53 and p21 pathways and upregulating CDK4 and cyclin A [28]. The L213I/S in the S region, which tended to be higher in rt269I HBV strains than in rt269L (*q* = 0.054), has been reported to be related to disease progression in chronic patients) [44]. The rt269L signature type, I68T in the S region, which is significantly prevalent in the rt269L versus rt269I type (*q* = 0.004), has been reported to be an HCC-independent risk factor in chronic Chinese patients with genotype C infections [45].

#### 3.5.2. X Region

Since the HBV X region includes not only the X protein coding region but also the regulatory region, mutation in the X region can simultaneously affect both the transactivating activity of HBxAg and the regulation of HBV replication [46]. We found a total of four types of signature NS mutations in the X ORF (V/L5M, T36P/S/A, S101P/A and V131I). Of these, three NS mutations, V/L5M, S101P/A and V131I, and one, T36P/S/A, were rt269I and rt269L signature types, respectively. V/L5M, which tended to be higher in rt269I strains than in rt269L strains, has been reported to be significantly related to HCC progression and HBeAg-negative serostatus in chronic patients with genotype C2 infections) [24]. V131I also leads to one of the double mutations in the overlapping basal core promoter (1764 G to A), which is significantly prevalent in rt269I strains versus rt269L (*q* = 0.004) and has also been reported to be related to HBsAg-negative serostatus and HCC progression in chronic patients [47]. T36P/S/A, a rt269L signature type that is more prevalent in rt269I strains than in rt269L strains (*q* < 0.001), has been reported to be higher in HCC patients, which enables the escape of the host immune response, possibly due to its location at the B epitope [48].

#### 3.5.3. PreC/C region

Mutations in the preC/C region can affect HBeAg serostatus and antigenicity, HBV nucleocapsid structure and stability, and the packaging of pregenomic RNA into the nucleocapsid [49]. Furthermore, HBcAg is the principal target of the host cell-mediated immune response [50,51] of which mutations can induce persistent HBV infections) [14,52]. We found a total of three types of signature NS mutations in the preC/C region (one in the preC region: preC-W28Stop, two in the C region: C-I97F/L (A2189T/C or C2191T mutation) and C-Q182K/* (C2444A/T or A2445G mutation)). All the three were rt269I signature types. The preC-W28Stop, known as a preC mutation at 1896 nt, leads to premature termination of HBeAg, resulting in HBeAg-negative infection in chronic patients with its mutation, of which the frequency was significantly higher in rt269I strains than in rt269L strains (*q* < 0.001). A number of studies have reported that preC-W28Stop, a preC 1896 mutation, is significantly related to liver disease progression [53,54]. The C-I97F/L in the C region, of which the frequency was significantly higher in rt269I strains than in rt269L strains (*q* = 0.047), is well known as the most frequently encountered HBcAg mutation, as mentioned in several studies [55,56] leading to an immature secretion phenotype due to defective nucleocapsid assembly. This can contribute to the progression of severe liver disease by failing to elicit a proper host immune response against HBV infection. Indeed, a previous study reported that C-I97F/L (A2189T/C or C2191T mutation) was the preC/C type found the most frequently in HCC patients in Korean chronic patients infected with genotype C2 [13,14]. C-Q182K/* (C2444A/T or A2445G mutation), of which the frequency tended to be higher in rt269I strains than in rt269L strains (*q* = 0.068), has been reported to be significantly related to HCC in Korean chronic patients infected with genotype C2 [13,14].

**Table 3:** Comparison of the signature nonsynonymous mutation rate between rt269L and rt269I.

Mutation Region			Amino Acid		Reference Strains		FDR Corrected *p*-Value (*q*-Value)		
					L Type (*n* = 115)	I Type (*n* = 119)		Function	Ref
preS1	I84T/M/L/V		Ile (I) Thr (T) Met (M) Leu (L) Val (V)		99 (86.09%) 9 (7.83%) 1 (0.87%) 1 (0.87%) 2 (1.74%)	82 (68.91%) 18 (15.13%) 3 (2.52%) 14 (11.76%) 2 (1.68%)	<0.001 ^c^	Transactivator domain, Occult infection	[17]
	A90T/V		Ala (A) Th r(T) Val (V)		84 (73%) 31(27%)	57(47.9%) 1 (0.8%) 61 (51.3%)	<0.001 ^c^		
preS2	F141L		Phe (F) Leu (L)		100 (86.96%) 12 (10.43%)	95 (79.83%) 24 (20.17%)	0.046 ^a^	HCC	[27]
S	sI68T		Ile (I) Thr (T)		81 (70.43%) 34 (29.57%)	103 (86.55%) 16 (13.45%)	0.004 ^b^	HCC	[57]
	sL213I/S		Leu (L) Ile (I) Ser (S)		110 (96.65%) 5 (4.35%) 0 (0.00%)	105 (88.24%) 13 (10.92%) 1 (0.84%)	0.054	Disease progression	[58]
Terminal protein	D16A/E/N/V		Asp (D) Ala (A) Glu (E) Asn (N) Val (V)		110 (95.65%) 1 (0.87%) 2 (1.74%) 1 (0.87%) 1 (0.87%)	105 (88.24%) 3 (2.52%) 4 (3.36%) 3 (2.52%) 4 (3.36%)	0.054	N-terminal helix1 mutation	[59]
Spacer	S314P		Ser (S) Pro (P)		78 (67.83%) 37 (32.17%)	50 (42.01%) 69 (57.98%)	<0.001 ^c^		
	F321L		Phe (F) Leu (L)		79 (68.70%) 36 (31.30%)	51 (42.86%) 68 (57.14%)	<0.001 ^c^		
Reverse transcriptase	rtN139K/H		Asn (N) Lys (K) His (H)		112 (98.26%) 0(0.00%) 1 (1.74%)	112 (94.96%) 2 (1.68%) 5 (4.20%)	0.066	Pretreatment mutation	[60]
	rtM204I/V		Met(M) Ile (I) Val (V)		111(96.52%) 4 (3.48%) 0 (0.00%)	103 (86.55%) 14 (11.76%) 2 (1.68%)	0.009 ^b^	resistance to LMV, LdT, ADV, TNF	[61,62]
	rtI224V		Ile (I) Val (V)		110 (95.65%) 4 (3.48%)	106 (89.08%) 13 (10.92%)	0.042 ^a^	Pretreatment mutation	[60]
RNaseH	D828A/V		Asp (D) Ala (A) Val (V)		105(91.30%) 6(5.22%) 4 (3.48%)	88 (73.95%) 5 (4.20%) 26 (21.85%)	0.001 ^b^	putative catalytic site mutants	[63]
HBx	V/L5M		Val (V) Leu (L) Met (M)		98 (85.22%) 6 (5.22%) 10 (8.7%)	92 (77.31%) 6 (5.04%) 21 (17.65%)	0.054	Independent predictors of HCC survival, miRNA binding site	[11]
	T36P/S/A		Thr (T) Pro (P) Ser (S) Ala (A)		57 (50%) 22 (19.3%) 22 (19.3%) 13 (11.4%)	89 (75.42%) 7 (5.93%) 9 (7.63%) 13 (11.02%)	<0.001 ^c^	B cell epitope	[64]
HBx	S101P/A		Ser (S) Ala (A) Pro (P)		103(89.6%) 10 (8.7%) 1 (0.90%)	78 (65.5%) 18 (15.1%) 23 (19.3%)	<0.001 ^c^		
	V131I		Val (V) Ile (I)		30 (26.79%) 82(73.21%)	14 (11.76%) 105 (88.24%)	0.004 ^b^	HCC	[65]
Precore	G1896A		Trp (W) Stop		84 (73.04%) 31 (26.96%)	61 (51.26%) 58 (48.74%)	<0.001 ^c^	HBeAg-negative serostatus	[14]
Core	A2189T/C, C2191T		Ile (I) Phe (F) Leu (L)		75 (65.22%) 5 (4.35%) 35 (30.43%)	62 (52.10%) 4 (3.36%) 53 (44.54%)	0.047 ^a^	HBeAg-negative serostatus, HCC-related HBcAg mutation	[14,66]
	C2444A/T, A2445G		Gln (Q) Tyr (Y) His (H) Stop		111 (96.52%) 1 (0.87%) 0 (0.00%) 3 (2.61%)	107 (89.92%) 2 (1.68%) 4 (3.36%) 6 (5.04%)	0.068	HCC-related HBcAg mutation	[14]

^a,b,c^ Statistically significant after Benjamini–Hochberg FDR (^a^ *q* < 0.05, ^b^ *q* < 0.01, ^c^ *q* < 0.001).

#### 3.5.4. Pol Region

Preexisting HBV Pol mutations in treatment-naïve patients are related to potential drug resistance, progression of liver disease, such as HCC or cirrhosis, and final clinical outcomes of drug treatment [57]. Furthermore, several studies reported that patients with chronic HBV carrying preexisting Pol mutations had significantly decreased serum baseline HBV DNA loads and HBeAg seronegative infections [67,68]. We found a total of seven types of signature NS mutations in the Pol region (three types in RT, one type in the terminal protein (TP) region, two types in the spacer region and one type in the RNaseH region), of which all seven (rtN139K/H, rtM204I/V and rtI224V in RT, D16A/E/N/V in TP, S314P and F321L in Spacer and D828A/V in the RNaseH region) were rt269I signature types. Three NS mutation types in the RT region have been reported to belong to preexisting reverse transcriptase (RT) mutations related to nucleos(t)ide analog (NAr) resistance. rtM204I, a YMDD motif mutation whose frequency was significantly higher in rt269I than in rt269L (*q* = 0.009), can lead to resistance to various drugs, including lamivudine (LMV), and it is the most frequently encountered preexisting NAr mutation worldwide [62,69]. Of note, of the seven types, the following three rt269I signature types, rtN139K/H, rtM204I/V and rtI224V, have been reported to be NAr RT mutation types related to clinical severity, including HCC or liver cirrhosis [68,70,71,72,73]

## 4. Discussion

**Figure 5:**
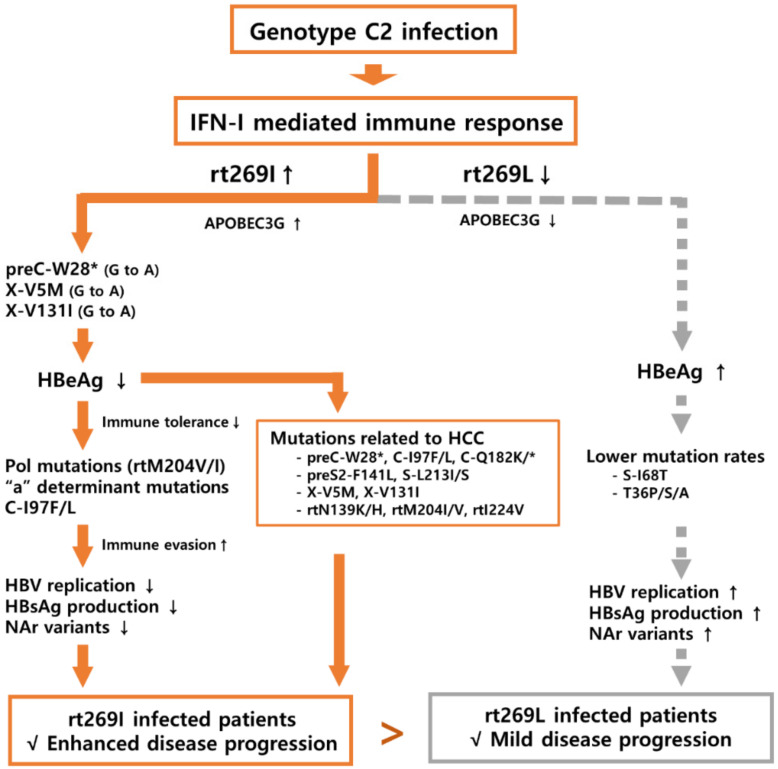
Schematic presentation. Schematic image showing that rt269I type versus rt269L type is more prone to overall genome mutations, particularly in the Pol and MHR regions in genotype C2 infections. The signature nonsynonymous mutations can contribute to the decreased level of HBV DNA replication, HBeAg secretion, NAr variants, and HCC occurrence. Finally, rt269I type versus rt269L type can lead to liver disease development.

HBV genotype C2 has two polymorphisms, L and I, at the 269th codon of Pol RT, distinct from other genotypes having one I type alone, that can provide in part a likely explanation into unique virological and clinical traits found in genotype C2 infections such as higher virulence, higher mutation frequency, or lower response to IFN-I therapy [74,75] Indeed, we have previously reported that the rt269I type of genotype C2, but not genotype A, can lead to enhanced IFN-I production in infected hepatocytes, resulting in HBeAg seronegative infections via IFN-I mediated 1896 preC G to A mutation and lowered HBV DNA or HBsAg production [76], suggesting the possibility of being more prone to mutation within the HBV genome in patients with rt269I of genotype C2 versus rt269L. IFN-I could mediate antiviral action via upregulating APOBEC3G that hypermutates the HBV genome [77]. In addition, in the previous study, we found that rt269I enhanced IFN-I-mediated APOBEC3G signaling in infected hepatocytes (Figure 5), which could lead to the higher mutation frequency in G to A mutation in preC and BCP in patients with rt269I [76]. Therefore, in this study, we address this issue and explore rt269L or rt269I signature mutations by analyzing mutation frequency and types from complete HBV genome sequences randomly selected from an HBV database—HBVdb (http://hbvdb.ibcp.fr (accessed on 14 March 2021)).

In this study, we found that overall mutation rates of HBV nucleotides and amino acids are significantly higher in rt269I HBV strains than in rt269L strains (Table 1 and Figure 2). Of note, dN and d of amino acid in Pol region or RT region were significantly higher in rt269I than in rt269L strains, suggesting that rt269I versus rt269L strains are more prone to HBV mutations, particularly NS mutations in HBV Pol region (Table 1 and Figure 3). Given the previous finding that HBV Pol mutations are significantly associated with lower HBV replication or HBeAg negative serostatus [68], higher HBV mutations found in rt269I types may be due to enhanced host immune responses after conversion into HBeAg seronegative status, which could result in persistent infections in chronic patients with genotype C2 infections.

Our data also showed that NS mutations within MHR or “a” determinant in the S region, a major target against host humoral immune response, are significantly higher in rt269I HBV strains than in rt269L strains (Table 2 and Figure 4), suggesting that rt269I versus rt269L strains are more likely to generate immune or vaccine escape variants, also contributing persistent infections of rt269I strains in chronic patients of genotype C2 infections.

In this study, we found a total of 19 NS signature mutation types, of which all 17 types, except for two types specific to rt269L (I68T in S region and T36P/S/A in X), were rt269I signature type. In rt269I signature NS mutations, several types, including preC-W28Stop (1896 preC mutation) and two types in the X region, V/L5M and V131I, are strongly related to HBeAg seronegative infections, which can lead to enhanced persistent rt269I type HBV infections by increasing mutation frequencies within the HBV genome, particularly in the Pol region, perhaps because of a host immune response. This could resultantly contribute to rt269I-type liver disease progression during the natural course of HBV genotype C2 infections (Figure 5). Indeed, we found several rt269I signature mutation types related to lower HBV replication (I84T/M/L/V in PreS1, preC-W28Stop, V/L5M in X region, C-I97F/L in C region and rtM204I/V in RT region) or liver disease progression (three types in preC/C region (preC-W28Stop, C-I97F/L and C-Q182K/*), F141L in PreS2, L213I/S in S region, three types in X region (V/L5M, T36P/S/A and V131I), three types in Pol region (rtN139K/H, rtM204I/V and rtI224V)) (Table 3 and Figure 5).

The present study has several major limitations. First, HBV genome sequences were used from only an HBV database, HBVdb (http://hbvdb.ibcp.fr (accessed on 14 March 2021)), and differences between distinct clades within sub-genotype C2 were not considered in this study. To elucidate further details regarding molecular epidemiologic traits of rt269L and rt269I strains in the chronic patients of genotype C2, a larger sample size of HBV sequences from multiple HBV databases reflecting diverse geo-ethnic traits and distinct clades within sub-genotype C2 has to be analyzed in future study. Second, although some meaningful differences between rt269L and rt269I strains were found from *t*-test-based analysis, the difference could not reach statistical significance in multiple comparisons using Benjamini–Hochberg correction. Given that the variation between samples was rather large [39], it could be supplemented by further study using larger sample size of HBV sequences.

In conclusion, our data showed that rt269I type versus rt269L type is more prone to overall genome mutations, particularly in the Pol region in genotype C2 infections, and is more prevalent in signature NS mutations related to lowered HBV DNA replication, HBsAg and HBeAg secretion, potential NAr variants and HCC, perhaps via IFN-I mediated enhanced inflammation. Our data suggest that rt269I could contribute to liver disease progression via the generation of immune escape or potential NAr variants in chronic patients of genotype C2 (Figure 5).

## Figures and Tables

**Table 1:** Comparison of genetic diversity within the hepatitis B virus (HBV) full genome, PreS, S, X, PreC/C and Pol regions between rt269L and rt269I.

	L Type (*n* = 115)	I Type (*n* = 119)	*p*-Value from *t*-Test
Number of nucleotide variants			
HBV full genome (/3,215 bp)	38.03 ± 13.65	42.34 ± 15.17	0.0234 *
Number of amino acid variants			
HBV full genome (/1,613 aa)	23.43 ± 10.75	27.68 ± 12.39	0.0056 **
d (10^−2^ substitution/site) (nucleotide level)			
preS SHB pol RT X preC/C	1.39 ± 1.59 0.58 ± 0.37 1.09 ± 0.46 0.97 ± 0.40 1.24 ± 0.61 1.38 ± 0.86	1.71 ± 1.78 0.64 ± 0.45 1.24 ± 0.50 1.03 ± 0.48 1.28 ± 0.60 1.56 ± 0.80	0.1559 0.2522 0.0154 * 0.3216 0.6752 0.0989
d (10^−2^ substitution/site) (amino acid level)			
preS SHB pol RT X preC/C	1.60 ± 2.50 1.10 ± 0.95 1.26 ± 0.79 0.88 ± 0.67 2.19 ± 1.43 1.64 ± 1.56	2.23 ± 3.29 1.29 ± 1.01 1.60 ± 0.86 1.06 ± 0.72 2.34 ± 1.30 1.66 ± 1.29	0.0989 0.1494 0.0019 **^a^ 0.0465 * 0.3796 0.9128
dN (10^−2^ substitution/site)			
preS SHB pol RT X preC/C	0.81 ± 1.40 0.52 ± 0.45 0.61 ± 0.42 0.42 ± 0.32 1.04 ± 0.70 0.82 ± 0.81	1.11 ± 1.74 0.62 ± 0.49 0.78 ± 0.45 0.51 ± 0.35 1.10 ± 0.62 0.82 ± 0.65	0.1449 0.1142 0.0044 **^a^ 0.0327 * 0.4405 0.9903
dS (10^−2^ substitution/site)			
preS SHB pol RT X preC/C	2.78 ± 2.23 0.68 ± 0.55 2.21 ± 0.74 2.30 ± 1.05 1.72 ± 1.05 2.45 ± 1.62	3.22 ± 2.07 0.69 ± 0.65 2.31 ± 0.86 2.26 ± 1.23 1.68 ± 0.90 2.94 ± 1.62	0.1188 0.9058 0.2855 0.8048 0.7902 0.0212 *

d, genetic distance; dS, the number of synonymous substitutions per synonymous site; dN, the number of non-synonymous substitutions per non-synonymous site; the significant values from *t*-test were shown in boldface and marked with asterisk (* *p* < 0.05, ** *p* < 0.01); ^a^ Statistically significant after Benjamini–Hochberg false discovery rate (FDR) (^a^ *q* < 0.05).

**Table 2:** Comparison of genetic diversity within MHR and “a” determinant regions between rt269L and rt269I.

	L Type (*n* = 115)	I Type (*n* = 119)	*p*-Value from *t*-Test
Number of nucleotide variants			
a determinant (/72 bp) MHR (/213 bp)	0.29 ± 0.59 1.14 ± 1.10	0.50 ± 0.92 1.31 ± 1.38	0.0311 * 0.2935
Number of amino acid variants			
a determinant (/24 aa) MHR (/71 aa)	0.23 ± 0.45 0.24 ± 0.54	0.33 ± 0.68 0.41 ± 0.69	0.2182 0.0399 *
dN (10^−2^ substitution/site)			
a determinant MHR	0.07 ± 0.38 0.48 ± 0.53	0.38 ± 1.24 0.67 ± 0.87	0.0109 * 0.0497 *
dS (10^−2^ substitution/site)			
a determinant MHR	0.99 ± 2.58 1.39 ± 1.48	1.14 ± 2.36 1.26 ± 1.45	0.6425 0.5065

Abbreviations: d, genetic distance; dS, the number of synonymous substitutions per synonymous site; dN, the number of non-synonymous substitutions per non-synonymous site; the significant values from *t*-test were shown in boldface and marked with asterisk (* *p* < 0.05).

## Data Availability

All the data produced here is available and can produced when required.

## Supplementary Materials

The following are available online at https://www.mdpi.com/2076-2607/9/3/601/s1, Table S1. All the accession numbers of each sequence and the origin of the virus.
