# Supplementary material for: rt269I Type of Hepatitis B Virus (HBV) Polymerase versus rt269L Is More Prone to Mutations within HBV Genome in Chronic Patients Infected with Genotype C2: Evidence from Analysis of Full HBV Genotype C2 Genome
Source: Microorganisms. 2021 Mar 15;9(3):601. doi: 10.3390/microorganisms9030601 (PMC7999911; doi:10.3390/microorganisms9030601)
Supplement: Supplementary file 1 [file microorganisms-09-00601-s001.pdf]

I type	Country	L type	Country
**AB900116**	Japan	AB900115	Japan
**EU939543**	China	EU939538	China
**EU939544**	China	EU939539	China
**EU939545**	China	EU939540	China
**EU939546**	China	EU939542	China
**EU939549**	China	EU939548	China
**EU939551**	China	EU939550	China
**EU939555**	China	EU939562	China
**EU939561**	China	EU939567	China
**EU939563**	China	EU939571	China
**EU939564**	China	EU939572	China
**EU939565**	China	EU939578	China
**EU939566**	China	EU939582	China
**EU939568**	China	EU939586	China
**EU939569**	China	EU939593	China
**EU939570**	China	EU939597	China
**EU939579**	China	EU939604	China
**EU939584**	China	EU939607	China
**EU939585**	China	EU939609	China
**EU939587**	China	EU939613	China
**EU939588**	China	EU939614	China
**EU939589**	China	EU939615	China
**EU939590**	China	EU939616	China
**EU939591**	China	EU939640	China
**EU939592**	China	EU939647	China
**EU939594**	China	EU939651	China
**EU939595**	China	EU939652	China
**EU939596**	China	EU939653	China
**EU939600**	China	EU939654	China
**EU939601**	China	EU939658	China
**EU939603**	China	FJ386574	China
**EU939605**	China	FJ386576	China
**EU939610**	China	FJ386578	China
**EU939611**	China	FJ386581	China
**EU939612**	China	FJ386585	China
**EU939617**	China	FJ386587	China
**EU939618**	China	FJ386588	China
**EU939619**	China	FJ386595	China
**EU939644**	China	FJ386602	China
**EU939646**	China	FJ386604	China
**EU939648**	China	FJ386605	China
**EU939649**	China	FJ386606	China
**EU939655**	China	FJ386609	China
**EU939656**	China	FJ386611	China
**EU939657**	China	FJ386613	China
**EU939659**	China	FJ386618	China
**FJ386575**	China	FJ386620	China
**FJ386577**	China	FJ386626	China
**FJ386579**	China	FJ386627	China
**FJ386580**	China	FJ386631	China
**FJ386586**	China	FJ386632	China
**FJ386589**	China	FJ386633	China
**FJ386591**	China	FJ386635	China
**FJ386592**	China	FJ386638	China
**FJ386596**	China	FJ386639	China
**FJ386597**	China	FJ386644	China
**FJ386598**	China	FJ386647	China
**FJ386601**	China	FJ386649	China
**FJ386603**	China	FJ386652	China

I type	Country	L type	Country
**FJ386607**	China	FJ386659	China
**FJ386614**	China	FJ386662	China
**FJ386617**	China	FJ386671	China
**FJ386619**	China	FJ386672	China
**FJ386624**	China	FJ386678	China
**FJ386625**	China	FJ386679	China
**FJ386628**	China	FJ386685	China
**FJ386630**	China	FJ562218	China
**FJ386637**	China	FJ562221	China
**FJ386651**	China	FJ562225	China
**FJ386653**	China	FJ562226	China
**FJ386657**	China	FJ562227	China
**FJ386661**	China	FJ562228	China
**FJ386663**	China	FJ562232	China
**FJ386670**	China	FJ562233	China
**FJ386673**	China	FJ562238	China
**FJ386687**	China	FJ562243	China
**FJ386689**	China	FJ562248	China
**FJ562235**	China	FJ562264	China
**FJ562239**	China	FJ562265	China
**FJ562241**	China	FJ562266	China
**FJ562242**	China	FJ562272	China
**FJ562244**	China	FJ562279	China
**FJ562245**	China	FJ562280	China
**FJ562249**	China	FJ562281	China
**FJ562251**	China	FJ562282	China
**FJ562252**	China	FJ562283	China
**FJ562255**	China	FJ562284	China
**FJ562258**	China	FJ562285	China
**FJ562261**	China	FJ562294	China
**FJ562267**	China	FJ562287	China
**FJ562268**	China	FJ562295	China
**FJ562269**	China	FJ562299	China
**FJ562273**	China	FJ562301	China
**FJ562274**	China	FJ562306	China
**FJ562275**	China	FJ562308	China
**FJ562276**	China	FJ562313	China
**FJ562288**	China	FJ562315	China
**FJ562291**	China	FJ562318	China
**FJ562292**	China	FJ562319	China
**FJ562293**	China	FJ562325	China
**FJ562304**	China	FJ562327	China
**FJ562305**	China	FJ562329	China
**FJ562307**	China	FJ562332	China
**FJ562310**	China	FJ562335	China
**FJ562314**	China	FJ562337	China
**FJ562317**	China	KF485389	Argentina
**FJ562320**	China	KF485390	Argentina
**FJ562324**	China	KJ410510	Hong Kong
**FJ562330**	China	KM359441	Argentina
**FJ562333**	China	KM999991	China
**FJ562334**	China	KP017270	South Korea
**FJ562340**	China	KP017271	Malaysia
**GU385774**	China	KP017272	Taiwan
**HQ622095**	Brazil	KP027477	China
**JN315779**	South Korea	AB368296	Japan
**KJ173426**	China		
**KJ410521**	Hong Kong		
**KP017269**	Japan		
**AB900099**	Japan		
